# Evaluation of the Hindgut Microbiota and Volatile Fatty Acid Profile of Steers Fed Finishing Feedlot Ration Supplemented with or Without Calcium Gluconate

**DOI:** 10.3390/microorganisms14040802

**Published:** 2026-04-01

**Authors:** Osman Y. Koyun, Evann E. Rowland, Jeferson M. Lourenco, Kenneth E. Griswold, Joseph J. Baloyi, Francis L. Fluharty, T. Dean Pringle, Alexander M. Stelzleni, R. Lawton Stewart, Todd R. Callaway

**Affiliations:** 1 Department of Animal and Dairy Science, University of Georgia, Athens, GA 30602, USA; yasirkoyun@hotmail.com (O.Y.K.); evann.rowland25@uga.edu (E.E.R.); jefao@uga.edu (J.M.L.); joseph.baloyi@univen.ac.za (J.J.B.); ffluharty@uga.edu (F.L.F.); dpringle@uga.edu (T.D.P.); astelz@uga.edu (A.M.S.); lawtons@uga.edu (R.L.S.); 2 Micronutrients Inc., Selko, Indianapolis, IN 46231, USA; ken.griswold@selko.com; 3 Department of Animal Science, University of Venda, Thohyandou 0950, South Africa; 4 Institute of Food and Agricultural Sciences, University of Florida, Quincy, FL 32351, USA

**Keywords:** Angus steers, prebiotic, calcium gluconate, butyrate, hindgut microbiota

## Abstract

Growing Angus steers (n = 20) were blocked by weight and randomly assigned to one of two treatment groups: Control group (CON, n = 10) fed a feedlot ration ad libitum, or a ruminally protected hydrogenated fat-embedded calcium gluconate (HFCG) treatment group (HFCG, n = 10), which was fed the control ration top-dressed at 16 g/head/day for 55 days. During the slaughter process, digesta samples were collected from the cecum, colon, and rectum. Acetate concentrations were greater in the cecal and rectal digesta of steers (*p* ≤ 0.05) in the HFCG treatment group. Propionate concentrations were greater in the cecal, colonic, and rectal (*p* ≤ 0.05) digesta of steers in the HFCG treatment group. Butyrate concentrations were greater (*p* = 0.098) in the colon digesta of steers in the HFCG treatment group; however, they were not different in the cecal and rectal digesta. To determine the microbial composition within each section of the hindgut, DNA was extracted, and 16S rRNA gene sequencing was performed. Data were analyzed using a General Linear Model with dietary treatment as the main effect. Species richness in the cecum, colon, and rectum was not different between treatments. *Erysipelotrichaceae*, *Peptostreptococcaceae*, and *Atopobiaceae* abundances were increased (*p* ≤ 0.05) in the cecal bacterial community of steers in the HFCG group, while a significant decrease (*p* ≤ 0.05) in *Rikenellaceae* and *Muribaculaceae* abundances was recorded within the same bacterial community. In the colon bacterial community of steers in the HFCG group, *Ruminococcaceae* and *Muribaculaceae* abundances were elevated (*p* ≤ 0.05), while there was a reduction (*p* ≤ 0.05) in *Lachnospiraceae*, *Erysipelotrichaceae*, *Atopobiaceae*, and *Peptostreptococcaceae* abundances. *Paeniclostridium*, *Romboutsia*, and *Turicibacter* abundances were increased (*p* ≤ 0.05) in the cecal bacterial community of steers in the HFCG group, while there was a decrease (*p* ≤ 0.05) in *Rikenellaceae_RC9 _gut_group* abundance within the same bacterial community. In the colon microbiota of steers in the HFCG group, *Turicibacter* abundance was decreased (*p* ≤ 0.05). Supplementing growing steers with HFCG impacted some members of the bacterial populations, which have important roles in gut homeostasis and health, along with the formation of beneficial end-products in the gastrointestinal tract.

## 1. Introduction

Ruminant animals have a gastrointestinal tract (GIT) that is colonized by a complex ecosystem of bacteria, fungi, protozoa, and archaea, which degrade feedstuffs, enabling the animal to harvest energy and protein for growth [1,2,3]. In particular, the rumen microbial ecosystem provides the ruminant animal the ability to harvest energy from forages and other feedstuffs, yet this adaptability comes at the cost of lower feed efficiency than monogastric animals [4,5]. While cattle productivity primarily depends on the symbiotic microbial fermentation of feedstuffs in the rumen, a secondary fermentation occurs in the hindgut (e.g., cecum and colon), which provides further metabolizable energy to the animal, and the fecal microbiota activity has been linked with improving the feed efficiency of cattle [6,7,8]. The dietary energy produced from the hindgut fermentation in the form of volatile fatty acids (VFAs, e.g., acetate, butyrate, and propionate) contributes to the energy available for ruminants to utilize for maintenance, growth, and production [5,9,10]. Among these predominant VFAs that have beneficial effects for animals, butyrate has received particular attention from researchers. Butyrate is a vital VFA produced in the GIT via microbial fermentation of dietary carbohydrates that are ingested by the host and is associated with a healthy gut in the host [2,11,12].

In ruminants, butyrate is mainly produced in the rumen, where it is absorbed rapidly; however, it is also present in high concentrations in the cecum and colon due to secondary hindgut fermentation [11,12]. Butyrate is the preferred energy source for colonocytes, accounting for approximately 70% of the total energy consumption of the colonocyte [12]. The anti-inflammatory effect of butyrate provides a benefit to the host animal by minimizing the energy directed to the fight against diseases; therefore, it frees up that energy for host growth and development [13,14]. Moreover, butyrate stimulates mucus production, increases cell proliferation in the GIT, strengthens the intestinal barrier by enhancing tight junction assembly, and affects nutrient, mineral, and water absorption from the intestinal lumen [12,15]. The use of butyrate as a dietary supplement in animals has been limited due to its volatility and offensive odor; however, butyrate salts (e.g., calcium, sodium, magnesium, or potassium salts), and encapsulated products as well as butyrate precursors have been developed and investigated to increase butyrate supply in the GIT of animals [11,12].

Gluconate is produced by a variety of microorganisms (e.g., bacteria and fungi) through the dehydrogenation of glucose via glucose oxidase, which naturally occurs in plants, fruits, wine, honey, and meat [16,17,18,19]. It has desirable characteristics for use in foods and feeds because it is odorless, non-volatile, non-corrosive, non-toxic, and soluble in water [16,17,18,20]. Classified as generally recognized as safe (GRAS) by the United States Food and Drug Administration (FDA), gluconate and its alkali salts (e.g., calcium gluconate and sodium gluconate) are used as bio-based additives in food and medicines [16,17,18,20]. When fed to monogastric species, gluconate has demonstrated a prebiotic effect, including escaping ruminal and abomasal degradation and small intestinal absorption and being available for microbial fermentation in the hindgut [21,22]. Gluconate enhanced animal performance through improving average daily gain [23,24], which was associated with changes in microbial VFA production in the host hindgut because gluconate is identified as a precursor for butyrate synthesis [25,26]; however, evidence in ruminant species in this regard is still scarce. Moreover, the majority of gluconate-fermenting bacteria in the hindgut are lactic acid bacteria (LAB) that produce mainly lactate, which is in turn converted to butyrate by acid—utilizing butyrate-producing bacteria [21,22,25,26]. This relationship between LAB and acid—utilizing butyrate-producing bacteria, and the fermentation pattern of gluconate in the ruminant hindgut, has yet to be elucidated.

In the first published study conducted in ruminant species, calcium gluconate supplementation increased milk fat yield in lactating dairy cattle [27]. The method of calcium gluconate delivery demonstrated a differential impact on animal performance, with post-ruminal infusion of calcium gluconate increasing milk fat yield and energy-corrected milk yield in lactating dairy cattle [28,29]; however, when directly dosed in the rumen, gluconate addition reduced weight gain and milk yield [29]. Supplementing dairy cattle with a hydrogenated fat-embedded calcium gluconate (HFCG; designed to reach the hindgut for calcium gluconate delivery) increased or tended to increase milk fat yield and both fat- and energy-corrected milk yield [30,31,32]. Another recent study has confirmed the effectiveness of HFCG supplementation at increasing milk yield, protein content and yield, and lactose yield in lactating cattle [33]. Feeding HFCG to growing lambs does not increase butyrate concentration in the intestine, the absorptive surface area, functionality, or size of the GIT [34]. In addition, while both post-ruminal infusion of calcium butyrate and HFCG affected the molar proportion of acetate and propionate in the colon, only post-ruminal infusion of calcium butyrate stimulated ruminal short-chain fatty acid (SCFA) absorption for growing beef heifers [35]. There is little information on the utilization of gluconate in the hindgut of ruminant animals, and the impact of gluconate supplementation on the VFA profile and microbiota in the hindgut of ruminant animals is not fully understood. Therefore, the objective of the study was to evaluate the impact of feeding a ruminally protected hydrogenated fat-embedded calcium gluconate supplement (HFCG; a prebiotic targeting the hindgut) on hindgut microbiota and VFA profile using Angus beef steers as a gut model. We hypothesized that feeding HFCG to Angus beef steers would modulate the hindgut bacterial community and enhance VFA production in the hindgut.

## 2. Materials and Methods

### 2.1. Experimental Design

All animal care followed guidelines approved by the University of Georgia’s Animal Care and Use Committee (UGA AUP A2020 02-017-Y1-A0). The study started on April 14 and ended on 5 June 2020. Angus steers with a starting weight of approximately 448 kg (9.6 kg SEM) that were calved and raised at the Eatonton Beef Research Unit, Eatonton, GA, USA, were utilized in the study. All steers (n = 20) were reared in a pasture-based system from 9 months of age until enrollment in the present study at 13 months of age, before being transported to the feedlot in Eatonton. Upon arrival at the feedlot, steers were acclimated to the Calan gate system (Calan Broadbent Feeding System; American Calan, Northwood, NH, USA) and to the control feedlot finishing rations fed (Table 1) during the adaptation phase (−49 d), before being fed either the control (CON; n = 10) ration or HFCG [Selko^®^ Lactibute, Micronutrients, Indianapolis, IN, USA; a ruminally protected hydrogenated fat-embedded calcium gluconate] treatment ration (HFCG; n = 10) for 55 or 62 days based on slaughter group. The current study utilized a completely randomized design. Steers in each treatment were blocked by weight and randomly assigned to two slaughter groups. 6 steers from the CON group and 4 steers from the HFCG group were slaughtered during the first slaughter date (d 56), and the alternate 4 steers from the CON group and 6 steers from the HFCG group were slaughtered on the second slaughter date (d 63). The two slaughter weeks were required due to COVID limitations on the processing facility procedures. All steers were fed their respective treatment rations until the day prior to slaughter. All steers always had ad libitum access to water.

### 2.2. Rations: Control and HFCG Treatments

The ration (Table 1) was prepared at a commercial feed mill (Godfrey’s Feed, Madison, GA, USA) and delivered to the Eatonton Beef Research Facility. Each day, the HFCG supplement was top-dressed (16 g/hd/d) and mixed by hand in each HFCG group steer’s Calan feed bunk. Steers were fed ad libitum with a target of 10% refusals.

### 2.3. Gastrointestinal Sample Collection and pH Measurements

At slaughter, digesta contents from each hindgut compartment (e.g., cecum, colon, and rectum) were aseptically collected (two subsamples per compartment, 5–10 g/subsample) for analysis and stored in sterile 40 mL plastic tubes. Additionally, digesta pH from each section was measured immediately at the site using an Orion pH meter (Thermo Fisher Scientific, Waltham, MA, USA) and was frozen at −20 °C until later analysis. Each sample was aseptically transported on ice from the Meat Science Teaching and Research Laboratory to the pilot laboratory (less than 10 min away from the sample collection site).

### 2.4. Volatile Fatty Acid (VFA) Analysis in Hindgut Digesta

Concentrations of VFA in gastrointestinal digesta from each sampled hindgut compartment were analyzed [36]. Briefly, 3 g of each sample was placed into 15 mL conical tubes and diluted with 9 mL of distilled water, and mixed prior to subsampling. Tubes were vortexed for 30 s, and 2.0 mL of the mixture was transferred to centrifuge tubes, which were then centrifuged at 10,000× *g* for 10 min; 1 mL of the resulting supernatant was aliquoted into new centrifuge tubes, along with 0.2 mL of a meta-phosphoric acid solution (25% weight/volume), and samples were frozen overnight. Samples were thawed and centrifuged at 10,000× *g* for 10 min. The supernatant was transferred into polypropylene tubes and mixed with ethyl acetate in a 2:1 ratio of ethyl acetate to supernatant. Tubes were vortexed for 15 s and allowed to settle for 5 min before 0.5 mL of the supernatant was transferred to screw-thread vials for VFA analysis using a Shimadzu GC-2010 Plus gas chromatograph (Shimadzu Corporation, Kyoto, Japan) equipped with a flame ionization detector and a capillary column (Zebron ZB-FFAP; 30 m × 0.32 mm × 0.25 μm; Phenomenex Inc., Torrance, CA, USA). The sample injection volume was set as 1.0 μL, and helium was used as the carrier gas. The column temperature was initially set at 110 °C and gradually increased to 200 °C. Injector and detector temperatures were set at 250 and 350 °C, respectively. The samples’ peak heights were compared to standards to determine the concentrations of VFAs in the digesta samples.

### 2.5. DNA Extraction and Sequencing

Deoxyribonucleic acid (DNA) was extracted from the GIT samples following previously described procedures, which used a combination of mechanical and enzymatic methods to optimize DNA extraction [36,37,38]. Briefly, this procedure uses 0.33 g of sample placed in 2-mL Lysing Matrix E tubes (MP Biomedicals LLC, Irvine, CA, USA), which are homogenized in a FastPrep 24 Instrument (MP Biomedicals LLC, Irvine, CA, USA) to disrupt the cells. Enzymatic inhibition was achieved by using InhibitEX Tablets (QIAGEN, Venlo, The Netherlands), and DNA elution and purification were conducted using an automated robotic workstation (QIAcube; QIAGEN, Venlo, The Netherlands). Determination of DNA concentration and purity in the resulting eluate was performed spectrophotometrically using the Synergy H4 Hybrid Multi-Mode Microplate Reader in conjunction with the Take3 Micro-Volume Plate (BioTek Instruments Inc., Winooski, VT, USA). Samples with a minimum concentration of 10 ng/μL of DNA were frozen at −20 °C until shipping. Samples that failed to meet these requirements were rejected and subjected to a new DNA extraction cycle.

### 2.6. DNA Library Preparation and Bioinformatics Analysis

Extracted DNA samples were transported frozen to LC Sciences (Houston, TX, USA) for 16S rRNA gene sequencing of the V3–V4 hypervariable regions. Library preparation included PCR replications using the forward: S-D-Bact-0341-b-S-17 (5′-CCTACGGGNGGCWGCAG-3′); and reverse: S-D-Bact-0785-a-A-21 (5′-GACTACHVGGGTATCTAATCC-3′) primer pair [36]. The amplified DNA was then sequenced using an Illumina MiSeq instrument (Illumina Inc., San Diego, CA, USA). Demultiplexed paired-end sequences were imported into QIIME 2 Version 2024.1 [39] for further processing. Non-biological nucleotides were removed, and sequences were denoised, dereplicated, and chimera-filtered using the DADA2 plugin 12 January 2024 access [40]. Taxonomic classifications were assigned to the sequences using a pre-trained Naïve Bayes classifier based on the SILVA database [37], and reads were classified by taxon using the fitted classifier. Samples underwent rarefication to 4780 sequences per sample prior to computing alpha diversity. The following alpha diversity indices were computed: Observed Amplicon Sequence Variants (ASV), Chao1 Index, Shannon Diversity Index, Simpson Diversity Index, and Good’s Coverage Index. The relative bacterial abundance was quantified at the phylum, family, and genus levels.

### 2.7. Statistical Analysis

Statistical analysis was conducted using MINITAB^®^ Statistical Software (2021). The study was a completely randomized design using the individual steer as the experimental unit. An ANOVA (General Linear Model; GLM) was constructed for VFA concentrations as well as microbiome results (individual microbial taxa and alpha diversity indices) and included treatment as the main effect. Least square means were separated using the Fisher LSD Method. Significance was declared at *p* ≤ 0.05, and tendency was determined as 0.05 < *p* ≤ 0.10.

## 3. Results

### 3.1. VFA Concentrations and pH in Hindgut Digesta

Acetate concentrations were greater in the cecal (79.7 mM vs. 61.8 mM; *p* = 0.01) and rectal (52.7 mM vs. 32.0 mM; *p* = 0.02) digesta of steers in the HFCG group (Table 2). There was a tendency (*p* = 0.08) towards an increased acetate concentration (56.4 mM vs. 35.6 mM) in the colon digesta of steers in the HFCG group (Table 2). Propionate concentrations were greater in the cecal (28.97 mM vs. 17.86 mM; *p* = 0.02), colon (21.2 mM vs. 11.3 mM; *p* ≤ 0.05), and rectal (18.6 mM vs. 9.09 mM; *p* = 0.01) digesta of steers in the HFCG group (Table 2). There was a tendency towards a greater butyrate concentration (9.13 mM vs. 4.9 mM; *p* = 0.098) in the colon digesta of steers in the HFCG group; however, butyrate concentrations in the cecal (14.1 mM vs. 13.1 mM) and rectal (6.82 mM vs. 6.85 mM) digesta were not different between the groups (Table 2). Isobutyrate concentrations were not different between groups in any of the digesta collected from the hindgut. In both the cecal and rectal digesta of steers in the HFCG group, total VFA concentrations were (*p* = 0.01 and *p* = 0.02, respectively) greater than those of steers in the CON group (125.41 mM vs. 95.55 mM and 79.97 mM vs. 49.22 mM, respectively; Table 2). There was a tendency (*p* = 0.07) towards a greater total VFA concentration in the colon digesta of steers in the HFCG group (98.29 mM vs. 58.97 mM). The ratio of acetate to propionate (A:P) in the cecal, colon, or rectal digesta of all steers was not significantly different between treatment groups. Mean pH of hindgut digesta at slaughter is presented in Table 3. Cecal digesta pH in the CON group was significantly greater when compared to the cecal digesta pH in the HFCG group (6.32 vs. 5.95; *p* = 0.03). Colon digesta pH tended to be greater in the CON group (6.31 vs. 5.97; *p* = 0.07). No significant difference between treatments was observed in the rectal digesta pH (6.28 vs. 6.11; *p* = 0.39).

### 3.2. Bacterial Richness and Diversity

**Figure 1:**
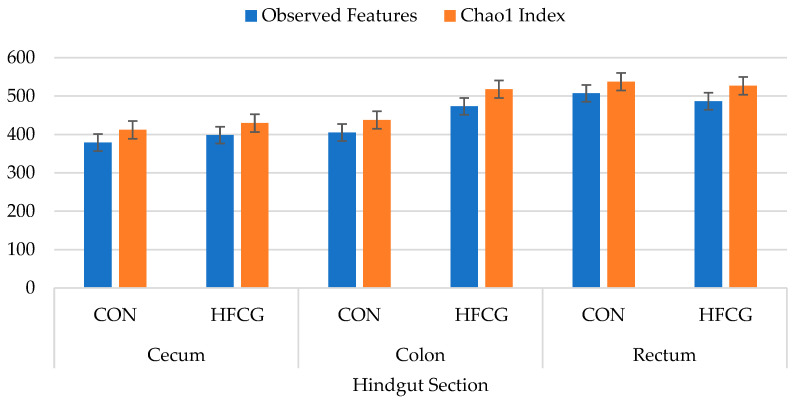
Effect of HFCG treatment on bacterial richness (indicated by Observed Features [ASV] and Chao1 Index) in the cecum, colon, and rectum digesta of steers (n = 10/treatment) from CON or HFCG groups. The calcium gluconate supplement (16 g/hd/d) was added as a top-dressing to each HFCG group ration daily.

The alpha diversity indices used in this study were: Number of observed features (ASV), Chao1 Index, Shannon Diversity Index, Simpson Diversity Index, and Good’s Coverage Index. Although we observed numerical differences, there were no significant differences between treatment groups in species richness (in either the Chao1 Index or the number of observed features) of bacterial communities in the cecum, colon, or rectum (Figure 1). The greatest numbers of Observed Features and the Chao1 Index were recorded in the rectum bacterial community of steers in the CON group (Figure 1, Supplementary Table S1). Shannon and Simpson diversity indices were numerically greater in all bacterial communities in all hindgut compartments of steers in the HFCG group (Supplementary Table S1).

### 3.3. Hindgut Microbiota Composition at Phylum Level

*Firmicutes*, *Bacteroidetes*, *Actinobacteria*, *Spirochaetes*, and *Proteobacteria* were the predominant phyla in the bacterial community of sampled hindgut sections (Table 4). *Firmicutes* and *Actinobacteria* abundances were increased (*p* = 0.007 and *p* = 0.012, respectively), and *Bacteroidetes* abundance was decreased (*p* = 0.004) in the cecal microbiota of steers in the HFCG group compared to the CON group. *Actinobacteria* abundance was decreased (*p* = 0.018), and *Bacteroidetes* abundance tended to increase (*p* = 0.075) in the colon microbiota of steers in the HFCG group compared to the CON group. No significant difference was observed between treatment groups in the abundance of the predominant phyla in the rectum; however, *Spirochaetes* abundance tended (*p* = 0.074) to increase in the rectum microbiota of steers in the HFCG group. *Firmicutes*:*Bacteroidetes* ratio was elevated (*p* ≤ 0.05) in the cecal microbiota and decreased (*p* = 0.008) in the colon microbiota of steers in the HFCG group.

### 3.4. Hindgut Microbiota Composition at Family Level

**Figure 2:**
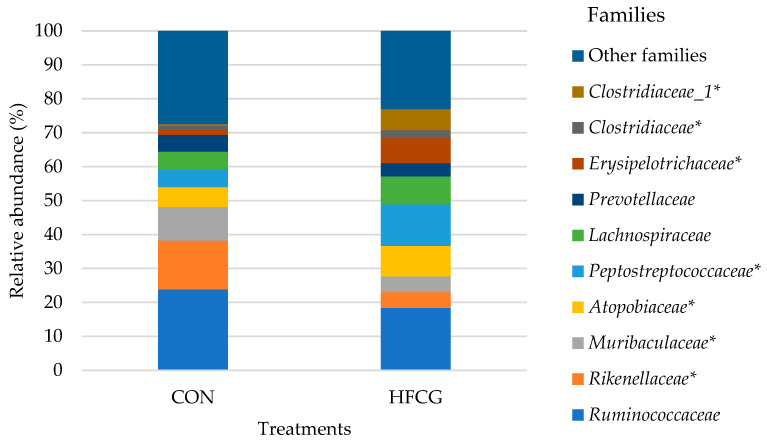
Effect of HFCG treatment on the cecal microbiota of steers (n = 10/treatment from CON or HFCG groups) at the family level. The calcium gluconate supplement (16 g/hd/d) was added as a top-dressing to each HFCG group ration daily. (*) refers to the statistical significance of *p* ≤ 0.05.

**Figure 3:**
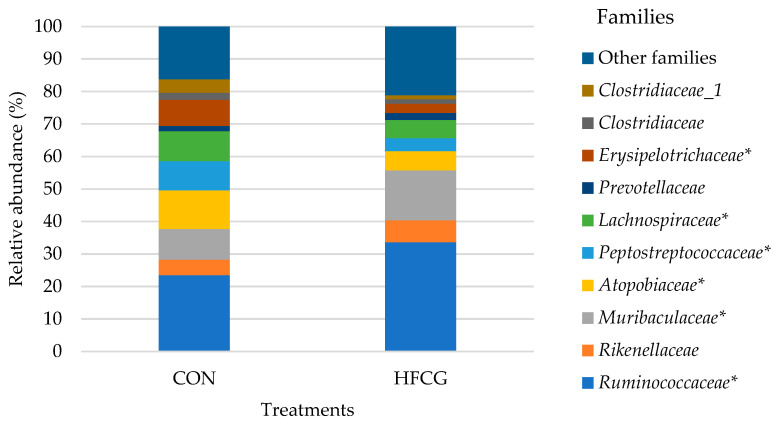
Effect of HFCG treatment on the colonic microbiota of steers (n = 10/treatment from CON or HFCG groups) at the family level. The calcium gluconate supplement (16 g/hd/d) was added as a top-dressing to each HFCG group ration daily. (*) refers to the statistical significance of *p* ≤ 0.05.

Microbial composition at the family level for the cecum, colon, and rectum of all steers is presented in detail in Supplementary Table S2. *Prevotellaceae*, *Lachnospiraceae*, *Ruminococcaceae*, *Erysipelotrichaceae*, *Atopobiaceae*, *Rikenellaceae*, *Muribaculaceae*, *Clostridiaceae*, *Clostridiaceae_1*, and *Peptostreptococcaceae* were some of the predominant families in the bacterial community of sampled hindgut compartments of all steers (Figure 2 and Figure 3). The abundance of *Erysipelotrichaceae*, *Peptostreptococcaceae*, *Clostridiaceae*, *Clostridiaceae_1*, and *Atopobiaceae* was increased (*p* = 0.003, *p* = 0.011, *p* = 0.001, *p* = 0.009, *p* = 0.001, and *p* = 0.012, respectively) in the cecal bacterial community of steers in the HFCG group (Figure 2). A significant decrease (*p* = 0.002 and *p* = 0.021, respectively) in the abundance of *Rikenellaceae* and *Muribaculaceae* was recorded within the same bacterial community (Figure 2). Additionally, *Spirochaetaceae* abundance tended to decrease (*p* = 0.069) in the cecal bacterial community of steers in the HFCG group (Supplementary Table S2). The abundance of *Ruminococcaceae* and *Muribaculaceae* was elevated (*p* = 0.019 and *p* = 0.004, respectively) in the colon bacterial community of steers in the HFCG group (Figure 3), while there was a reduction (*p* = 0.033, *p* = 0.015, *p* = 0.023, and *p* = 0.002, respectively) in the abundances of *Lachnospiraceae*, *Erysipelotrichaceae*, *Atopobiaceae*, and *Peptostreptococcaceae* within the same bacterial community (Figure 3). Furthermore, *Rikenellaceae* abundance was increased (*p* = 0.045) in the rectal bacterial community of steers in the HFCG group (Supplementary Table S2).

### 3.5. Hindgut Microbiota Composition at Genus Level

**Figure 4:**
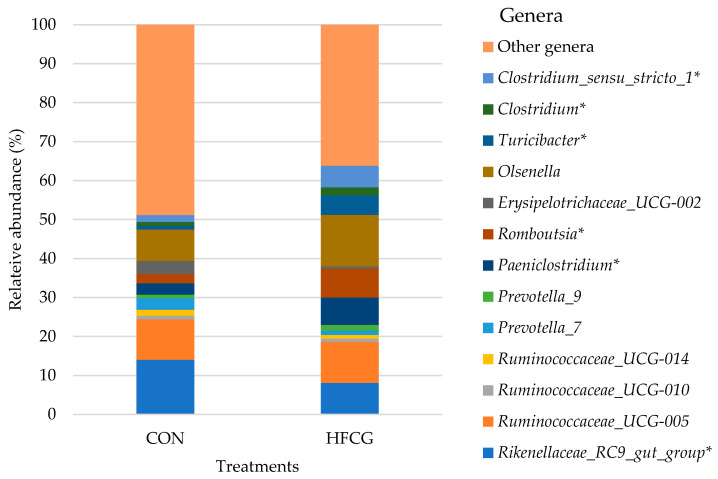
Effect of HFCG treatment on the cecal microbiota of steers (n = 10/treatment from CON or HFCG groups) at the genus level. The calcium gluconate supplement (16 g/hd/d) was added as a top-dressing to each HFCG group ration daily. (*) refers to the statistical significance of *p* ≤ 0.05.

**Figure 5:**
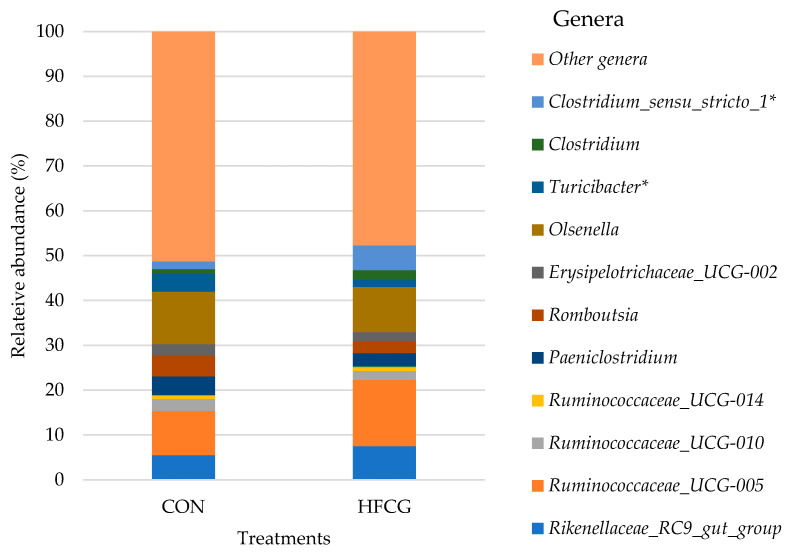
Effect of HFCG treatment on the colonic microbiota of steers (n = 10/treatment from CON or HFCG groups) at the genus level. The calcium gluconate supplement (16 g/hd/d) was added as a top-dressing to each HFCG group ration daily. (*) refers to the statistical significance of *p* ≤ 0.05.

The lowest taxonomic level at which the samples in this study were classified was the genus level, and the predominant bacterial genera detected in the cecum, colon, and rectum of all steers are presented in detail in Supplementary Table S3. Resolution in this study was not sufficient to assign ASVs consistently at the species level. *Ruminococcaceae_UCG-005*, *Ruminococcaceae_UCG-010*, *Ruminococcaceae_UCG-014*, *Rikenellaceae_RC9 _gut_group*, *Paeniclostridium, Romboutsia*, *Erysipelotrichaceae_UCG-002*, *Olsenella*, *Turicibacter*, *Prevotella_7*, and *Prevotella_9* were found to be the predominant genera in the cecum and colon microbiota of all steers (Figure 4 and Figure 5). The abundance of *Paeniclostridium*, *Romboutsia*, and *Turicibacter* was increased (*p* = 0.007, *p* = 0.005, and *p* = 0.001, respectively) in the cecal bacterial community of steers in the HFCG group, while there was a decrease (*p* = 0.022) in the abundance of *Rikenellaceae_RC9 _gut_group* within the same bacterial community (Figure 4). Additionally, *Clostridium_sensu_stricto_1* and *Clostridium* abundances were elevated (*p* = 0.019 and *p* = 0.012, respectively) in the cecal bacterial community of steers in the HFCG group (Figure 4). In the colon microbiota of steers in the HFCG group, *Turicibacter* and *Clostridium_sensu_stricto_1* abundances were decreased (*p* = 0.014 and *p* = 0.037, respectively; Figure 5).

## 4. Discussion

Cattle productivity heavily depends on the microbial fermentation of feedstuffs in the rumen; however, a secondary microbial fermentation occurs in the hindgut, which has been associated with improving the feed efficiency of cattle [6,7,41]. Substrates that are partially digested by enzymes in the rumen and small intestine are further broken down in the cecum and colon by a specialized consortia of microorganisms that is mostly comprised of bacteria [2,9,42,43]. Additional dietary energy derived from this hindgut microbial fermentation in the form of VFAs (e.g., acetate, butyrate, and propionate) is considered a significant contributor to energy availability in ruminants throughout all stages of production [3,9,43]. Among these major VFAs, butyrate is a crucial one as it (i) has an anti-inflammatory effect on the host [13,14], (ii) stimulates mucus production, (iii) strengthens the epithelial structural integrity in the GIT, (iv) affects the dynamics of nutrient, mineral, and water absorption in the GIT [12,15], and (v) is the preferred energy source for colonic epithelial cells, accounting for approximately 70% of the total energy consumption of the colonocytes [2,11,12]. In ruminant species, although butyrate is mainly produced in the rumen (approximately 8–10 mM), it is also found in high concentrations in the lumen of the large intestine, especially in the cecum and colon, due to the secondary hindgut fermentation [12]. Butyrate salts and encapsulated products, as well as butyrate precursors, have been developed and investigated to increase butyrate supply in the GIT of animals [11,12].

Gluconate is a precursor for butyrate synthesis [25,26] and has demonstrated a prebiotic effect when fed to monogastric species. Gluconate can survive the gastrointestinal passage and be available for microbial fermentation in the hindgut [21,22] and enhance animal performance through improving average daily gain [23,24]. Improved ADG was associated with changes in microbial VFA production in the host hindgut [25,26]. Unfortunately, there is a lack of information on the utilization of gluconate in the hindgut of ruminant animals, and the impact of gluconate supplementation on the hindgut VFA profile and microbiota in ruminants is not well understood.

Increasing use of Next Generation Sequencing (NGS) technologies has enabled researchers to gain more insight into the members and activities of the gastrointestinal microbiota of ruminants [7,44,45]. Therefore, understanding the rumen microbiota has garnered interest because of its substantial impact on animal health and productivity; however, the microbiota harbored in the remainder of the GIT has not received the same interest. Each compartment of the ruminant GIT deals with different fermentable substrates and presents different environmental conditions; therefore, the microbiota composition within each compartment changes from one to another. In general, the use of prebiotic compounds in ruminants has been challenging due to the diverse catabolic activity of ruminal microbially-produced carbohydrate active enzymes (CAZymes); however, protecting carbohydrates from microbial degradation can pass these compounds to the hindgut [44,46,47].

### 4.1. Cecal Microbiota Composition and VFA Profile

Acetate, propionate, as well as total VFA concentrations were significantly greater in the cecum of steers in the HFCG group in the present study (Table 2). Additionally, cecal digesta pH of steers in the CON group was significantly greater than that of steers in the HFCG group (Table 3). These results suggest that the microbial fermentation of gluconate (as a prebiotic source) by the cecum microbiota might have resulted in increased concentrations of certain VFAs and a lower pH in the cecal digesta of steers in the HFCG group in the current study.

Gluconate has been described as a precursor for butyrate synthesis in the hindgut of non-ruminant animals [22,26]. The effect of gluconate on in vitro growth response and metabolism of swine cecal microflora and on animal growth performance, intestinal wall morphology, and intestinal microflora was investigated [24]. Compared with the control group, gluconate tended to improve the average daily gain (ADG) of the piglets, yet cecal morphology and microbiota did not show any difference among treatments [24]. After 24 h of in vitro cecal fermentation, acetate, propionate, and butyrate concentrations were increased by gluconate [24]. Digesta pH in the small intestine and cecum as well as bacterial populations in both cecum and rectum, digesta of piglets were not affected by sodium gluconate supplementation [23]. Increasing the dietary supply of HFCG to growing lambs linearly increased the proportion of acetate in cecal digesta and linearly decreased the proportion of propionate in both cecal and colonic digesta [35]; however, it did not affect the butyrate concentrations in the hindgut. In the present study, calcium gluconate supplementation to the ration of steers did not increase butyrate concentration in the cecal digesta; instead, it elevated acetate and propionate concentrations in the cecum of steers in the HFCG group.

*Firmicutes*, *Bacteroidetes*, and *Actinobacteria* phyla were predominant in the cecum microbiota of all steers in the current study, corroborating previous studies where these phyla were commonly found in the hindgut microbiota of varied ruminant species under different experimental settings [8,10,43,48]. The abundance of *Firmicutes* and *Actinobacteria* was elevated while *Bacteroidetes* was decreased in the cecal microbiota of steers in the HFCG group compared to the CON treatment group; therefore, the *Firmicutes* to *Bacteroidetes* ratio was increased in the cecal microbiota, and the F:B ratio has been linked to increased dietary energy capture in mice [49,50,51] (Figure 2). The abundance of *Erysipelotrichaceae*, *Peptostreptococcaceae*, and *Atopobiaceae* (belonging to *Actinobacteria*) was significantly increased in the cecal bacterial community of steers in the HFCG group. A significant decrease in the abundance of *Rikenellaceae* and *Muribaculaceae* was recorded within the same bacterial community.

In the present study, *Lachnospiraceae* was abundant in the cecal digesta of steers regardless of the ration composition, and its abundance was numerically increased (from 5.12 to 8.28%) in the HFCG group. The abundant detection of *Lachnospiraceae* in the cecum of ruminant animals is a common finding reported by previous studies [6,9,42]. Several members of *Lachnospiraceae* are able to ferment pectin and break down other complex polysaccharides to VFAs (e.g., acetate, butyrate, and propionate) in the GIT environment, providing energy for the host [42,52,53]. Increased concentrations of acetate and propionate in the cecum of steers in the HFCG group can be associated with the (numerical) increase in the abundance of *Lachnospiraceae*. However, the exact role of *Lachnospiraceae* in the hindgut of ruminant species warrants further investigation.

Our data revealed that *Peptostreptococcaceae* was predominant in the cecal digesta regardless of the ration composition, and its abundance was increased (from 5.33 to 12.25%) due to the HFCG supplementation. Previous studies reported that *Peptostreptococcaceae* was also abundant in the cecum microbiota of ruminant animals investigated under different experimental conditions [54,55]. Some members of this family have been reported to play a vital role in feed digestion and are known as hyper-ammonia-producing bacteria [54,56]. However, the exact role of *Peptostreptococcaceae* in the GIT ecosystem(s), particularly in the cecum of ruminants, still warrants further investigation.

At the genus level, *Romboutsia* and *Paeniclostridium* represented the *Peptostreptococcaceae* family, and their abundances were also increased (from 2.36 to 7.47% and from 2.93 to 7.01%, respectively) in the cecal microbiota of steers in the HFCG group. *Romboutsia* as a genus has been linked to a healthy GIT mucosa in humans and functions as a microbial biomarker of polyps and/or colorectal cancer; therefore, its presence or elevated populations in the GIT may be considered beneficial to prevent intestinal dysbiosis [57]. *Romboutsia* species were also reported to be non-pathogenic and commensal in the GIT and capable of producing VFAs such as formate and acetate in the GIT of animals and of fermenting single amino acids [58,59,60]. It has been reported that *Romboutsia* may function as a candidate microbial biomarker of intestinal dysbiosis, as well as of coccidiosis in chickens, as its abundance in the chicken cecum declined post-infection caused by *Eimeria tenella* [60].

The addition of HFCG increased the crude fat content of the basal rations fed [31]. The impact of dietary fat on the abundance of *Erysipelotrichaceae* in the GIT of monogastric animals has been reported [61,62]. An increase in the abundance of this family was observed in the cecum and colon [61] microbiota of mice fed a high-fat diet. Additionally, a greater *Erysipelotrichaceae* abundance was reported in the cecal microbial community of steers with better average daily gain as well as dry matter intake [37]. The increase (from 1.52 to 7.44%) in the abundance of *Erysipelotrichaceae* in the cecal microbiota of steers in the HFCG group in our study may be attributed to the physical characteristics of the dietary supplement. At the genus level, *Turicibacter* and *Erysipelotrichaceae_UCG-002* represented the *Erysipelotrichaceae* family in the cecal microbiota of steers in the HFCG group (Figure 4). In particular, *Turicibacter* abundance was increased (from 0.93 to 4.98%; *p* > 0.05) at the expense of *Erysipelotrichaceae_UCG-002* and other members of the *Erysipelotrichaceae* family. *Turicibacter* represents the primary lactate producers in the large intestine [63] and was positively correlated with butyric acid production in rats fed high-fat diets [64].

*Clostridiaceae* and *Clostridiaceae_1* were other predominant bacterial families detected in the cecal microbiota of all steers, and their abundances were increased (from 1.17 to 2.33% and from 0.52 to 6.15%, respectively) due to the calcium gluconate supplementation (Supplementary Table S2). *Clostridiaceae* is a functionally diverse family comprised of members that are mostly commensal in the GIT and have important roles in the digestion of carbohydrates or proteins, altering their fermentation pathway depending on nutrient availability [64,65]. At the genus level, *Clostridium* and *Clostridium_sensu_stricto_1* represented the *Clostridiaceae* family, and their abundances were also increased (from 1.05 to 2.09% and from 1.73 to 5.52%, respectively) in the cecal microbiota of steers in the HFCG group (Supplementary Table S3). In animal as well as human GIT, *Clostridium* species are able to digest starch or indigestible/complex polysaccharides, and VFAs (e.g., acetate, propionate, and butyrate) that they produce provide benefits to the host by being used as energy sources by epithelial cells in the GIT [64,66].

### 4.2. Colonic Microbiota Composition and VFA Profile

The main function of the colon in ruminants is to absorb water from the digesta moving through it as well as vitamins produced by the microbiota harbored in this GIT compartment or originating from the digesta [3,9,52]. Moreover, the colon absorbs and utilizes the VFAs, especially the butyrate, generated from the post-ruminal degradation of cellulose, starch, and other partially digested feedstuffs [2,31]. In the present study, elevated concentrations of VFAs and a lower pH in the colon digesta can be attributed to the microbial fermentation of gluconate as a prebiotic agent in the colon. In particular, butyrate concentration tended to increase in the colon digesta of steers in the HFCG group, corroborating previous studies reporting gluconate as a butyrate precursor in the large intestine of non-ruminant animals [22,25,26]. While butyrate differences were not statistically significant, the role of butyrate on gut health/integrity has been increasingly demonstrated to play biologically impactful roles in animal and human health [11,15].

Increasing the dietary supply of HFCG to growing lambs linearly decreased the proportion of propionate in colonic digesta; however, it did not affect the butyrate concentrations [34]. While both post-ruminal infusion of calcium butyrate and HFCG affected the molar proportion of acetate and propionate in the colon, only post-ruminal infusion of calcium butyrate stimulated ruminal SCFA absorption for growing beef heifers [35]. Compared to the control group, both calcium butyrate and calcium gluconate treatments reduced the proportion of acetate and increased the proportion of propionate in the colonic digesta [35]. Acetate, butyrate, and total VFA concentrations tended to increase, and propionate concentration was significantly greater in the colon digesta of steers in the HFCG group (Table 2), and colon digesta pH tended to be greater in the CON group compared to the HFCG group (Table 3) in the present study. In the colon microbiota of steers in the HFCG group, the abundance of *Firmicutes* and *Actinobacteria* decreased while *Bacteroidetes* abundance tended to increase (Table 4).

*Bacteroidetes* in the GIT generally produce butyrate, which is an end-product of colon fermentation and plays a vital role in the gut health of the host [67]. Butyrate and other VFAs produced by the *Bacteroidetes* in the GIT are absorbed by the host, helping the host to gain energy from varied carbohydrate sources [67]. In the current study, acetate, butyrate, and propionate concentrations were greater (though not always reaching statistical significance), and total VFA concentration tended to increase in the colon digesta of steers in the HFCG group. Increased concentrations of VFAs in the colon digesta may be associated with the increase in the abundance of *Bacteroidetes,* as the breakdown of a variety of polysaccharides in the hindgut is the main biological function of this phylum as symbionts [67]. It is widely known that butyrate produced by the hindgut bacteria is a major energy source for the colon epithelium and regulates enterocyte proliferation and differentiation; thus, butyrate, as well as other VFAs, is vital to the GIT epithelium integrity and homeostasis [68,69]. *Bacteroidetes* was represented by *Prevotellaceae*, *Rikenellaceae*, and *Muribaculaceae* in the colon microbiota of steers regardless of the ration composition. Among these families in the colon microbiota of steers, *Muribaculaceae* abundance was significantly elevated in the HFCG group. *Muribaculaceae* was positively correlated with intramuscular fat deposition (i.e., marbling) in Angus steers [7]. In the present study, steers marbled well due to their diets earlier in their lives; however, the relationship with increased *Muribaculaceae* presence and/or abundance remains potentially important, as marbling is an economically important variable to beef producers.

At the family level, *Ruminococcaceae* (belonging to *Firmicutes*) abundance was significantly increased. In the current study, the elevated level of *Ruminococcaceae* can be correlated with increased VFA concentrations in the colon digesta, as this family has been reported as a major butyrate producer in the GIT [70]. The abundance of *Actinobacteria* was increased in the cecal microbiota of steers in the HFCG group, while there was a decrease in the abundance of this phylum in the colon microbiota. At the family level, the same trend was detected regarding *Atopobiaceae* abundance. Steers had a greater abundance of *Atopobiaceae* in the cecum microbiota but a lower abundance of this family in their colon microbiota. Certain *Actinobacteria* members, such as *Atopobiaceae,* are known as the producers of a variety of bioactive metabolites (e.g., VFAs) that are beneficial for the host’s gut health [71].

### 4.3. Rectal Microbiota Composition and VFA Profile

The digesta moving through the large intestine is exposed to a series of fermentative reactions along the GIT and reaches its final destination, which is the rectum that is connected to the anus, from where the remaining digesta/material is excreted as feces into the environment [2,3,48]. Bacteria found in feces have important roles in livestock farming, including nutrient cycling, producing gaseous compounds, and acting as a source for zoonotic pathogenic microorganisms as well as antibiotic-resistant bacteria [48]. In the current study, no significant difference was observed between treatment groups in the abundance of the predominant phyla. At the family level, *Rikenellaceae* abundance was increased in the rectal bacterial community of steers in the HFCG group (Supplementary Table S2).

Acetate, propionate, and total VFA concentrations were significantly greater in the rectal (fecal) digesta of steers in the HFCG group (Table 3); however, butyrate concentration and digesta pH were not different between the groups (Table 4). Similarly, supplementing dairy cattle with HFCG did not affect the pH or concentrations of acetate, propionate, or butyrate in the fecal digesta [31]. However, post-ruminal calcium gluconate infusion increased the concentration of fecal isobutyric acid in lactating dairy cattle [28].

### 4.4. Bacterial Richness and Diversity

Gut health is still not a clear term and is often described via a proxy measure: bacterial population richness and diversity. The host animal often depends on the energy produced by the microbial fermentation of feedstuff; therefore, any perturbation in bacterial populations can lead to changes in the predominant VFA (including lactate) produced and their gastrointestinal concentrations. Analysis of alpha diversity is a common approach to assess differences between biological samples, summarizing the structure of an ecological community with respect to its richness (e.g., number of taxonomic groups), diversity, and evenness (i.e., abundance distribution of the groups) or all [69,72]. The alpha diversity indices used in this study to obtain a comprehensive representation of all species in the samples are the following: Observed Features, Chao1 Index, Shannon Diversity Index, Simpson Diversity Index, and Good’s Coverage Index. Although there was a numerical difference, no significant difference was observed between treatment groups in species richness (in either the Chao1 Index or the number of Observed Features) of bacterial communities in the cecum, colon, or rectum. The greatest numbers of Observed Features and the Chao1 Index were recorded in the rectum bacterial community of steers in the CON group. Shannon and Simpson diversity indices, which are measures of species richness and evenness [73], were numerically greater in all bacterial communities in all GITs of steers in the HFCG group. Good’s coverage index provides an estimation of what percent of the total species is represented in a sample [72]. There was no difference in Good’s Coverage Index in the hindgut bacterial community of steers in the CON or HFCG group. The average Good’s Coverage for all samples was greater than 99%, indicating that the sequencing depth was adequate for a proper bacterial community analysis [72].

## 5. Conclusions

Supplementing the ration of growing steers with HFCG enhanced VFA production in the hindgut and had some impacts on the hindgut microbial ecosystem. HFCG supplementation led to shifts in bacterial composition through impacting populations of several bacterial genera (e.g., *Paeniclostridium*, *Romboutsia*, and *Turicibacter*), several of which have been reported to have vital roles in gut homeostasis and health and to play a role in the formation of end products that benefit the host animal’s energetic status (e.g., butyrate and propionate). Results provide novel insights into the potential of rumen—protected gluconate-based additives to modulate the hindgut microbiota in ruminants, a relatively underexplored site of fermentation. Although these results support the hypothesis that HFCG impacts hindgut microbial dynamics in favor of beneficial fermentative processes, further research is warranted to quantify corresponding changes in Volatile Fatty Acid concentrations (especially in butyrate, which is linked with gut integrity), monitor long-term impacts on growth performance, and evaluate possible interactions with host physiology.

## Figures and Tables

**Table 1:** Ration composition fed daily to both treatment groups (HFCG and CON; n = 10/treatment) of growing Angus steers and Nutrient Analysis (determined by Cumberland Valley Analytical Services, Waynesboro, PA, USA) of mixed ration fed to both treatment groups. The calcium gluconate supplement (16 g/hd/d) was added as a top-dress to the HFCG ration daily for each steer in the group. Values are presented on a DM basis. Steer rations were fed from d −49 through d 55 or 62.

Ingredient	% of Total Ration
Rolled corn	74.5
Corn gluten feed	10
Soyhull pellets	7
Soybean meal	6
Salt	1.8
Urea, 45%	0.5
Trace mineral salt	0.15
Vitamins A, D, and E	0.375
*Nutrient analyzed*	
	**% DM**
Moisture	14.4
Dry Matter (DM)	85.6
Crude Protein (CP)	14.3
Acid Detergent Fiber (ADF)	8.1
Neutral Detergent Fiber (NDF)	18.4
Crude Fat	3.2
Ash	4.7
Calcium	0.14
Phosphorous	0.37
Magnesium	0.16
Potassium	0.81
Total Digestible Nutrients	80.1

**Table 2:** Volatile fatty acid concentrations ([VFA], mM) in the digesta from each hindgut section of steers (n = 10/treatment) from the HFCG or CON groups. The calcium gluconate supplement (16 g/hd/d) was added as a top-dressing to each HFCG group ration daily. Individual *p*-values for treatment effect on VFA concentrations are indicated for each hindgut section below each column.

		Treatments			
Hindgut Section	VFAs	CON	HFCG	SEM ^1^	*p*-Value
**Cecum**	Acetate	61.8 ^b^	79.7 ^a^	3.52	0.01
	Propionate	17.86 ^b^	28.97 ^a^	2.43	0.02
	Butyrate	13.1	14.1	1.66	0.78
	Isobutyrate	0.69	0.61	0.08	0.38
	Total VFA	95.55 ^b^	125.14 ^a^	6.38	0.01
	Acetate:Propionate	3.46	2.75	1.14	0.147
**Colon**	Acetate	35.6	56.4	6.01	0.08
	Propionate	11.3 ^b^	21.2 ^a^	2.53	0.05
	Butyrate	4.9	9.13	1.28	0.098
	Isobutyrate	0.37	0.4	0.1	0.9
	Total VFA	58.97	98.29	9.66	0.07
	Acetate:Propionate	3.15	2.66	0.81	0.217
**Rectum**	Acetate	32 ^b^	52.7 ^a^	4.49	0.02
	Propionate	9.09 ^b^	18.6 ^a^	1.79	0.01
	Butyrate	6.85	6.82	1.26	0.99
	Isobutyrate	0.2	0.18	0.07	0.85
	Total VFA	49.22 ^b^	79.97 ^a^	6.91	0.02
	Acetate:Propionate	3.52	2.83	0.6	0.139

^ab^ Different superscripts within each row indicate differences (*p* ≤ 0.05) in VFA concentrations between treatments (CON vs. HFCG). ^1^ SEM: Standard error of the mean.

**Table 3:** Digesta pH in the cecum, colon, and rectum of steers (n = 10/treatment) from the CON or HFCG groups. The calcium gluconate supplement (16 g/hd/d) was added as a top-dressing to each HFCG group ration daily. Individual *p*-values for treatment effect on digesta pH are indicated for each hindgut section within each row.

	pH		
	Treatments		
Hindgut Section	CON	HFCG	*p*-Value
**Cecum**	6.32 ^a^	5.95 ^b^	0.03
**Colon**	6.31	5.97	0.07
**Rectum**	6.28	6.11	0.39

^ab^ Different superscripts within each row indicate differences (*p* ≤ 0.05) in pH between treatments (CON vs. HFCG).

**Table 4:** The predominant phyla in the cecum, colon, and rectum digesta of steers (n = 10/treatment) from the CON or HFCG groups. The calcium gluconate supplement (16 g/hd/d) was added as a top-dressing to each HFCG group ration daily. Individual *p*-values for treatment effect on predominant bacterial phyla are indicated for each hindgut section below each column.

		Treatments		
Hindgut Section	Indices	CON	HFCG	*p*-Value
**Cecum**	*Firmicutes*	50.39 ^b^	64.30 ^a^	0.007
	*Bacteroidetes*	36.20 ^a^	19.64 ^b^	0.004
	*Actinobacteria*	6.37 ^b^	10.03 ^a^	0.012
	*Spirochaetes*	0.37	0.30	0.551
	*Proteobacteria*	0.49	0.47	0.551
	*Firmicutes*:*Bacteroidetes*	1.38 ^b^	4.86 ^a^	0.001
	Other phyla	6.18	5.26	N/A
**Colon**	*Firmicutes*	64.24	56.26	0.109
	*Bacteroidetes*	19.16	29.08	0.075
	*Actinobacteria*	13.84 ^a^	6.63 ^b^	0.018
	*Spirochaetes*	1.96	2.48	0.811
	*Proteobacteria*	0.19	0.40	0.426
	*Firmicutes*:*Bacteroidetes*	3.33 ^a^	1.83 ^b^	0.008
	Other phyla	0.61	5.15	N/A
**Rectum**	*Firmicutes*	50.9	46.30	0.357
	*Bacteroidetes*	40.6	45.48	0.354
	*Actinobacteria*	5.98	2.01	0.306
	*Spirochaetes*	1.24	3.67	0.074
	*Proteobacteria*	0.71	0.89	0.513
	*Firmicutes*:*Bacteroidetes*	1.25	1.03	0.292
	Other phyla	0.59	1.65	N/A

^ab^ Means within a row without common superscripts are significantly different (*p* ≤ 0.05). N/A: Not available.

## Data Availability

The raw data supporting the conclusions of this article will be made available by the authors on request.

## Supplementary Materials

The following supporting information can be downloaded at: https://www.mdpi.com/article/10.3390/microorganisms14040802/s1, Table S1: Bacterial richness and alpha diversity indices of bacterial communities in the cecum, colon, and rectum digesta of steers (n = 10/treatment) from CON or HFCG groups. Table S2: The predominant families from bacterial compositions of the cecum, colon, and rectum digesta of steers (n = 10/treatment) from the CON or HFCG treatment groups. Table S3: The predominant genera in bacterial compositions of the cecum, colon, and rectum of steers (n = 10/treatment) fed CON or HFCG rations. Table S4: The predominant species in bacterial compositions of the cecum, colon, and rectum of steers (n = 10/treatment) fed CON or HFCG rations.

## Abbreviations

The following abbreviations are used in this manuscript:

VFA	Volatile fatty acids
GIT	Gastrointestinal tract
HFCG	Hydrogenated fat-embedded calcium gluconate
SCFA	Short-chain fatty acids
GRAS	Generally recognized as safe
FDA	Food and Drug Administration
LAB	Lactic acid bacteria
