# Supplementary material for: Evaluation of the Hindgut Microbiota and Volatile Fatty Acid Profile of Steers Fed Finishing Feedlot Ration Supplemented with or Without Calcium Gluconate
Source: Microorganisms. 2026 Apr 1;14(4):802. doi: 10.3390/microorganisms14040802 (PMC13118676; doi:10.3390/microorganisms14040802)
Supplement: Supplementary file 1 [file microorganisms-14-00802-s001.zip › microorganisms-3748591-supplementary.pdf]

SUPPLEMENTARY MATERIALS

Supplementary Table S1. Bacterial richness and alpha diversity indices of bacterial communities in the cecum, colon, and rectum digesta of steers (n = 10/treatment) from CON or HFCG groups. The calcium gluconate supplement (16g/hd/d) was added as a top-dressing to each HFCG group ration daily. Individual *p* – values for treatment effect on bacterial richness and alpha diversity measures are indicated for each hindgut section beside each row.

		**Treatments**		
**Hindgut Section**	**Indices**	**CON**	**HFCG**	***p*-value**
**Cecum**	Observed Features	379	398.5	0.966
	Chao1 Index	412	429.5	0.911
	Shannon Diversity Index	6.41	6.72	0.875
	Simpson Diversity Index	0.96	0.97	0.860
	Good's coverage	0.99	0.99	0.904
**Colon**	Observed Features	405.1	473.2	0.325
	Chao1 Index	437.8	518.1	0.370
	Shannon Diversity Index	6.61	7.04	0.585
	Simpson Diversity Index	0.97	0.98	0.896
	Good's coverage	0.99	0.99	0.364
**Rectum**	Observed Features	507.2	486.6	0.461
	Chao1 Index	537.5	526.9	0.564
	Shannon Diversity Index	7.01	7.17	0.739
	Simpson Diversity Index	0.97	0.98	0.410
	Good's coverage	0.99	0.99	0.492

Supplementary Table S2. The predominant families from bacterial compositions of the cecum, colon, and rectum digesta of steers (n = 10/treatment) from the CON or HFCG treatment groups. The calcium gluconate supplement (16g/hd/d) was added as a top-dressing to each HFCG group ration daily. Individual *p*– values for treatment effect on predominant bacterial genera are indicated for each gut section below each column.

		**Hindgut section**		
**Family**		**Cecum**	**Colon**	**Rectum**
Lachnospiraceae	CON	5.12	9.25[a]	6.77
	HFCG	8.28	5.61[b]	6.57
	p-value	0.259	0.033	0.619
Ruminococcaceae	CON	23.82	23.5[b]	26.45

	HFCG	18.37	33.6[a]	19.62
	p-value	0.205	0.019	0.110
Erysipelotrichaceae	CON	1.52[b]	8.07[a]	1.79
	HFCG	7.44[a]	2.82[b]	2.26
	p-value	0.003	0.015	0.688
Peptostreptococcaceae	CON	5.33[b]	9.0[a]	1.74
	HFCG	12.25[a]	4.0[b]	3.06
	p-value	0.001	0.002	0.689
Clostridiaceae	CON	1.17[b]	2.12	1.53
	HFCG	2.33[a]	1.38	0.69
	p-value	0.009	0.181	0.121
Clostridiaceae_1	CON	0.52[b]	4.16[a]	1.20
	HFCG	6.15[a]	1.28[b]	2.32
	p-value	0.001	0.001	0.847
Veillonellaceae	CON	3.81	0.17	0.60
	HFCG	0.37	0.24	1.86
	p-value	0.102	0.651	0.208
Prevotellaceae	CON	4.96	1.59	7.47
	HFCG	3.86	2.15	10.03
	p-value	0.869	0.573	0.138
Rikenellaceae	CON	14.36[a]	4.76	10.9[b]
	HFCG	4.63[b]	6.71	16.3[a]
	p-value	0.002	0.465	0.045
Bacteroidaceae	CON	0.88	1.74	2.61
	HFCG	0.97	1.72	1.44
	p-value	0.407	0.502	0.182
Muribaculaceae	CON	9.98[a]	9.44[b]	12.37
	HFCG	4.65[b]	15.4[a]	10.63
	p-value	0.021	0.004	0.551
Atopobiaceae	CON	5.80[b]	11.9[a]	4.03
	HFCG	8.99[a]	5.9[b]	1.71
	p-value	0.011	0.023	0.680
Spirochaetaceae	CON	3.24	2.20	2.47
	HFCG	0.50	2.36	3.65
	p-value	0.069	0.880	0.374
Acidaminococcaceae	CON	0.68	0.15	0.84
	HFCG	0.59	0.21	1.75
	p-value	0.868	0.985	0.395
F082	CON	0.06	0.11	0.08

	HFCG	0.28	0.02	1.98
	p-value	0.887	0.830	0.728
Christensenellaceae	CON	0.88	1.67	1.47
	HFCG	1.30	1.69	0.77
	p-value	0.218	0.780	0.129
Succinivibrionaceae	CON	0.12	0.02	0.27
	HFCG	0.26	0.07	0.88
	p-value	0.803	0.98	0.082
Tannerellaceae	CON	0.74	0.57	0.79
	HFCG	0.56	0.67	0.61
	p-value	0.876	0.229	0.872
Family_XII	CON	0.41	0.71	0.56
	HFCG	0.49	0.46	0.36
	p-value	0.746	0.053	0.114
Clostridiales_vadinBB60_group	CON	0.65	0.91	0.44
	HFCG	0.30	0.61	0.34
	p-value	0.719	0.884	0.413
Bifidobacteriaceae	CON	0.61	0.71	0.26
	HFCG	0.72	0.48	0.17
	p-value	0.810	0.144	0.414
Eggerthellaceae	CON	0.08[a]	0.36[a]	0.17
	HFCG	0.33[b]	0.21[b]	0.10
	p-value	0.012	0.021	0.113
p-251-o5	CON	2.93	0.81	1.28
	HFCG	0.24	2.22	3.52
	p-value	0.053	0.596	0.325
Other families	CON	12.33	6.1	13.93
	HFCG	16.14	10.16	9.34

[ab] Means within a column without common superscripts are significantly different ($p \leq 0.05$).

Supplementary Table S3. The predominant genera in bacterial compositions of the cecum, colon, and rectum of steers (n = 10/treatment) fed CON or HFCG rations. The calcium gluconate supplement (16g/hd/d) was added as a top-dressing to each HFCG group ration daily. Individual *p*– values for treatment effect on predominant bacterial genera are indicated for each gut section below each column.

		Hindgut section		
**Genus**		**Cecum**	**Colon**	**Rectum**
Ruminococcaceae_UCG-005	CON	10.34	9.78	10.90

	HFCG	10.50	14.77	9.00
	p-value	0.681	0.279	0.290
Ruminococcaceae_UCG-010	CON	0.93	2.73	2.06
	HFCG	0.86	2.00	1.32
	p-value	0.474	0.858	0.851
Ruminococcaceae_UCG-014	CON	1.55	0.75	1.74
	HFCG	0.98	0.87	0.81
	p-value	0.661	0.367	0.249
Erysipelotrichaceae_UCG-002	CON	3.38	2.50	0.99
	HFCG	0.52	2.01	0.42
	p-value	0.521	0.575	0.592
Turicibacter	CON	0.93[a]	3.95[a]	1.79
	HFCG	4.98[b]	1.61[b]	1.17
	p-value	0.001	0.014	0.387
Paeniclostridium	CON	2.93[a]	4.14	2.19
	HFCG	7.01[b]	3.00	1.62
	p-value	0.007	0.164	0.322
Romboutsia	CON	2.36[a]	4.67	2.17
	HFCG	7.47[b]	2.63	1.42
	p-value	0.005	0.151	0.487
Clostridium	CON	1.05[a]	2.11	1.37
	HFCG	2.09[b]	1.37	0.69
	p-value	0.012	0.176	0.208
Clostridium_sensu_stricto_1	CON	1.73[a]	5.33[a]	1.72
	HFCG	5.52[b]	2.65[b]	2.31
	p-value	0.019	0.037	0.896
*Eubacterium]_coprostanoligenes*_group	CON	4.26[a]	3.22[a]	4.19
	HFCG	1.79[b]	5.80[b]	2.02
	p-value	0.034	0.001	0.078
Prevotella_9	CON	0.93	0.15	1.23
	HFCG	1.48	0.14	2.49
	p-value	0.948	0.490	0.299
Prevotella_7	CON	2.93	0.01	1.52
	HFCG	1.04	0.00	1.71
	p-value	0.772	0.581	0.510
*Rikenellaceae*_RC9_gut_group	CON	14.05[a]	5.52	10.17
	HFCG	8.10[b]	7.50	15.90
	p-value	0.022	0.837	0.159
Bacteroides	CON	0.70	1.73	2.58

	HFCG	0.77	1.72	1.43
	p-value	0.374	0.472	0.161
Olsenella	CON	8.04	11.73	5.22
	HFCG	13.25	10.14	2.40
	p-value	0.176	0.562	0.341
Succiniclasticum	CON	0.31	0.02	0.82
	HFCG	1.46	0.01	3.03
	p-value	0.606	0.967	0.306
Treponema_2	CON	2.57	1.95	2.19
	HFCG	0.40	2.35	3.63
	p-value	0.061	0.878	0.324
Syntrophococcus	CON	0.79	0.71	1.28
	HFCG	3.31	0.53	0.66
	p-value	0.091	0.641	0.396
*Lachnospiraceae*_NK3A20_group	CON	1.05	0.24	0.39
	HFCG	0.95	0.20	1.09
	p-value	0.291	0.342	0.862
*Christensenellaceae*_R-7_group	CON	0.69	1.58	1.30
	HFCG	1.31	1.66	0.74
	p-value	0.170	0.697	0.194
Other genera	CON	38.48	37.18	44.18
	HFCG	26.21	39.04	46.14

[ab] Means within a column without common superscripts are significantly different ($p \leq 0.05$).

Supplementary Table S4. The predominant species in bacterial compositions of the cecum, colon, and rectum of steers (n = 10/treatment) fed CON or HFCG rations. The calcium gluconate supplement (16g/hd/d) was added as a top-dressing to each HFCG group ration daily. Individual *p*– values for treatment effect on predominant bacterial species are indicated for each gut section below each column.

		Hindgut section		
**Species**		**Cecum**	**Colon**	**Rectum**
Olsenella_provencensis	CON	3.89	4.99	1.96
	HFCG	4.61	3.25	1.17
	p-value	0.174	0.399	0.993
Olsenella_sp._G5(2011)	CON	1.42	2.77	1.79
	HFCG	5.08	4.23	0.46
	p-value	0.362	0.981	0.227
Olsenella_sp._Marseille-P8424	CON	0.35	0.86	0.34

	HFCG	0.79	0.72	0.17
	p-value	0.232	0.630	0.563
Olsenella_sp._Marseille-P8425	CON	0.23	0.74	0.32
	HFCG	0.63	0.45	0.08
	p-value	0.285	0.457	0.517
Bifidobacterium_sp._PFGE_14	CON	0.46	0.51	0.27
	HFCG	0.39	0.29	0.11
	p-value	0.890	0.446	0.677
Syntrophococcus_sp._BS-2	CON	0.25	0.53	0.21
	HFCG	0.33	0.39	0.16
	p-value	0.558	0.287	0.644
Duncaniella_muris	CON	0.16	0.05	0.17
	HFCG	0.13	0.81	0.65
	p-value	0.590	0.143	0.422
Eubacterium]_tenue	CON	2.93[b]	4.14	2.19
	HFCG	7.01[a]	3.00	1.62
	p-value	0.007	0.164	0.322
Muribaculum_sp.	CON	1.03	1.09[b]	1.56[a]
	HFCG	0.60	2.29[a]	0.81[b]
	p-value	0.257	0.001	0.041
Catonella_sp._feline_oral_taxon_009	CON	0.08	0.01	0.25
	HFCG	0.43	0.00	1.26
	p-value	0.905	0.560	0.443
Turicibacter_sanguinis	CON	0.16[b]	0.74[a]	0.22
	HFCG	0.57[a]	0.23[b]	0.16
	p-value	0.005	0.007	0.972
Bacteroides_rodentium	CON	0.01	0.95	0.95[a]
	HFCG	0.14	0.62	0.14[b]
	p-value	0.082	0.885	0.037
Clostridium_sp._enrichment_culture_clone_d-7	CON	0.73[b]	1.32	0.63
	HFCG	1.75[a]	0.74	0.18
	p-value	0.009	0.170	0.278
Prevotella_sp._AN_5135	CON	0.12	0.00	0.07
	HFCG	0.03	0.00	0.23
	p-value	0.832	0.911	0.483
uncultured_Olsenella_sp.	CON	0.45	0.64	0.27
	HFCG	0.67	0.40	0.22
	p-value	0.514	0.424	0.846
uncultured_Clostridium_sp.	CON	0.31	0.77	0.69

	HFCG	0.33	0.61	0.49
	p-value	0.488	0.600	0.404
uncultured_Succinivibrio_sp.	CON	0.12	0.01	0.16
	HFCG	0.58	0.02	0.54
	p-value	0.318	0.858	0.392
uncultured_Prevotella_sp.	CON	0.26	0.03	0.38
	HFCG	0.19	0.04	0.48
	p-value	0.778	0.892	0.487
uncultured_Turicibacter_sp.	CON	0.76[b]	3.21[a]	1.57
	HFCG	4.41[a]	1.38[b]	1.01
	p-value	0.001	0.019	0.320
uncultured_Bacteroides_sp.	CON	0.20	0.18	0.62
	HFCG	0.36	0.18	0.63
	p-value	0.500	0.921	0.950
Other species	CON	86.08	76.46	85.38
	HFCG	75.58	80.35	89.43

[ab] Means within a column without common superscripts are significantly different ($p \leq 0.05$).
